# Consumer input into research: the Australian Cancer Trials website

**DOI:** 10.1186/1478-4505-9-30

**Published:** 2011-06-26

**Authors:** Rachel F Dear, Alexandra L Barratt, Sally Crossing, Phyllis N Butow, Susan Hanson, Martin HN Tattersall

**Affiliations:** 1 Sydney Medical School, Room 391, Blackburn Building, D06, The University of Sydney NSW 2006, Australia; 2 Sydney School of Public Health, The University of Sydney, Sydney, Australia; 3 Cancer Voices NSW and Breast Cancer Action Group NSW, Sydney, Australia; 4 School of Psychology, The University of Sydney, Sydney, Australia; 5 Cancer Australia, Melbourne, Victoria, Australia; 6 Department of Medicine, The University of Sydney, Sydney, Australia

## Abstract

**Background:**

The Australian Cancer Trials website (ACTO) was publicly launched in 2010 to help people search for cancer clinical trials recruiting in Australia, provide information about clinical trials and assist with doctor-patient communication about trials. We describe consumer involvement in the design and development of ACTO and report our preliminary patient evaluation of the website.

**Methods:**

Consumers, led by Cancer Voices NSW, provided the impetus to develop the website. Consumer representative groups were consulted by the research team during the design and development of ACTO which combines a search engine, trial details, general information about trial participation and question prompt lists. Website use was analysed. A patient evaluation questionnaire was completed at one hospital, one week after exposure to the website.

**Results:**

ACTO's main features and content reflect consumer input. In February 2011, it covered 1, 042 cancer trials. Since ACTO's public launch in November 2010, until the end of February 2011, the website has had 2, 549 new visits and generated 17, 833 page views. In a sub-study of 47 patient users, 89% found the website helpful for learning about clinical trials and all respondents thought patients should have access to ACTO.

**Conclusions:**

The development of ACTO is an example of consumers working with doctors, researchers and policy makers to improve the information available to people whose lives are affected by cancer and to help them participate in their treatment decisions, including consideration of clinical trial enrolment. Consumer input has ensured that the website is informative, targets consumer priorities and is user-friendly. ACTO serves as a model for other health conditions.

## Background

The number of new cancer cases in Australia has more than doubled between 1982 and 2007 [1] and clinical trials are crucial to further progress cancer management and survival. Consumers, led by Cancer Voices NSW (CVN), have campaigned for many years for a national consumer-friendly website about cancer clinical trials which would enable consumers to undertake a comprehensive search for cancer trials recruiting anywhere in Australia and thus to be able to access information about relevant clinical trials. The need for easily accessible, understandable and authoritative information about treatment options, including clinical trials, for consumers has also been acknowledged by Australian Senate Inquiries [2] and in the National Service Improvement Framework for Cancer [3].

Low cancer patient participation in clinical trials may reflect lack of knowledge on the part of both doctors and patients about relevant trials that are recruiting [4,5]. Low recruitment rates may also be the result of difficulties faced by doctors in raising the option of clinical trial participation, and by patients in deciding whether to participate [6-8]. Evidence-based strategies are needed to encourage participation in cancer clinical trials [9]. Consumer advocates have been keenly aware of the need to increase clinical participation, seeing consumer-friendly information as key to achieving this goal.

Many patients also want involvement in decisions about their health care. For example, in a heterogeneous sample of cancer patients seeing an oncologist for the first time, 49% wanted shared decision making and 14% wanted to take the primary role [10]. To make informed health care choices, including whether to consent to participate in a clinical trial, patients need good information to weigh up their options. This need is reported as a priority by all health consumer advocacy groups.

Up to 60% of cancer patients use the internet to seek information about cancer, including clinical trials [11,12]. Internet-based services to search for and provide information about cancer clinical trials are widely available and consumer input has been used to make these websites more user-friendly [13,14]. Online clinical trial matching services have also been developed to help identify potential research participants [15-18]. The results of a number of surveys describe the willingness of people with cancer to search for information about cancer clinical trials on the internet [12,18-20]. The quality of information and the user-friendliness of cancer websites vary [21,22]. Clearly consumers should be able to access reliable, accurate, accessible and user-friendly web-based information to assist them to participate in decisions about their cancer care.

Effective consumer involvement can contribute to better health care and research and is widely recommended [23-27]. Involvement of relevant stakeholders in the development of websites is a much considered and researched aspect in the field of 'usability' of online health resources [28]. It is generally acknowledged that health communication benefits from materials tailored to the needs of the target audience rather than non-tailored material [29].

To meet the demand for a consumer-friendly cancer clinical trials website, the University of Sydney, Australian New Zealand Clinical Trials Registry (ANZCTR), CVN and Cancer Australia (CA) jointly developed the website called Australian Cancer Trials (ACTO) [30]. It is a not-for-profit national resource for people affected by cancer, doctors and researchers. It provides a search portal for clinical trials, information about clinical trials and trial participation and question prompt lists (QPLs)* (see footnote 1).

We aim to describe the involvement of consumers in the development of ACTO because it is an important resource that has the potential to be replicated for other health conditions. We also present the results of early patient use and reactions to the website.

## Methods

### Consumer consultation in the design of the Australian Cancer Trials website

The development of ACTO was informed by cancer consumers. The word "consumer" is used to mean people affected by cancer. Their involvement in this project meets the key components outlined in the model framework for consumer and community participation in health and medical research developed by the Consumers' Health Forum of Australia and the National Health and Medical Research Council (NHMRC) [23].

The lead consumer partner was CVN, which had called for a national consumer-friendly cancer clinical trials website for many years. CVN is an independent peak consumer organisation which works to provide a voice for people affected by cancer [31]. It is a reliable and trusted with a proven track record of involvement in research. For example, in partnership with the Cancer Council NSW it developed and participated in the Consumer Involvement in Research Project which provides a set of cancer consumer criteria which should be applied to research proposals [32] CVN also helped develop and contributes to the Cancer Consumer Advocacy Training Program [33].

The other major consumer partner was the National Consumer Advisory Group (NCAG) which was established in 2007 to help reduce the impact of cancer on Australians. Members of NCAG were recruited by CA [34], a federal government organisation, with the assistance of Cancer Voices Australia and Cancer Council Australia. It consisted of 15 consumers including representatives from the Cancer Voices network, Aboriginal and Torres Strait Islander representatives and three professionals selected to provide advice and guidance to consumers.

The impetus for the website began when SC, the chair of CVN, met with researchers from the University of Sydney at the end of 2006. At this time, it was proposed that the website should be developed using data about registered cancer trials from ANZCTR. In addition, the researchers proposed a formal evaluation of the impact of the website on doctor-patient discussions about trials and accrual rates. Subsequently, in early 2007, SC with representatives from the University of Sydney, ANZCTR and CA formed a research team to apply for a grant from the NHMRC to evaluate the website. In October 2007 it was announced that the grant was successful.

In her role as a research team principal investigator, SC contacted by email the Cancer Voices chairpersons from six Australian states and the Australian Capital Territory asking them to nominate existing cancer trials websites favoured by cancer consumers, key website design features and the information required for a consumer-friendly website. Three responses were received (from Cancer Voices South Australia, Victoria and New South Wales) and reported verbally at a meeting in October 2007. SC recorded their views in a summary report.

Concurrently during 2007, through NCAG's Cancer Voices membership, the development of a consumer-friendly website for CA was identified as a priority at the first meeting of NCAG. Subsequently, in consultation with ANZCTR, CA developed a background paper that included a table of current information collected about clinical trials from registrants and displayed on ANZCTR. From March until May 2007 the background paper was circulated to 50 consumers from across Australia from CA's network including young people affected by cancer. Consumers were requested to identify: 1) which of the data elements already available on the ANZCTR should be displayed on a consumer-friendly website and 2) what additional information would be required to assist consumers in making decisions about cancer clinical trials.

Twenty-one responses were received and collated into a report. In the report, two tables were provided: 1) a list of all the data elements consumers would need about each cancer trial to help make an informed decision and 2) additional consumer cancer items that needed to be collected by ANZCTR to satisfy consumer needs. In addition, the process of collecting and transferring data from ANZCTR to the consumer-friendly website was proposed.

The second NCAG meeting in October 2007 provided the opportunity to announce the NHMRC funding and for SC to present her summary report. An update was given by NCAG about their input into the website's development. To ensure that a consumer-friendly cancer clinical trials website would be achieved, CA entered into a partnership with ANZCTR and the research team. Work was progressed at the end of 2007 with the formation of a Cancer Clinical Trials Website Consumer Reference Group which had members from CVN, CA and NCAG.

This group met in January 2008 to further discuss 1) the terms from the clinical trial registries that should be extracted for display on ACTO; 2) the additional cancer items needed from researchers; and 3) a conceptual outline for ACTO. Draft website pages were designed and specific data elements to be included on each page were identified. Information fields were chosen that reflected the recommendations of the collated consumer views about what would be helpful for people to make informed decisions about participating in a cancer clinical trial. Consumers also listed what they considered to be essential elements of the search engine for the site. These findings were reported to the research team which, from 2008, proceeded with the website's development in consultation with CA, ANZCTR and a website development company.

Consumers consistently identified that a lay summary was an essential component in the display of information about each trial. As a result, consumers and researchers tested a method to develop standardised lay summaries. In June 2008, five members of the research team wrote lay summaries for 146 cancer trials already registered on the ANZCTR. Eleven Cancer Voices NSW consumer representatives, who had undertaken the Cancer Council NSW's Consumer Involvement in Research Training Program (a 2-day intensive training program in research methods and the application of lay criteria to evaluate research protocols) [35] critiqued these summaries. Their feedback indicated that the quality of the lay summaries was mixed. Their overwhelming recommendation was that the project should employ a professional medical writer to ensure that summaries would be consistent in style, phraseology and level of complexity.

Consumers agreed that the overall purpose of ACTO was that it should be a comprehensive source of cancer clinical trials in which consumers in Australia might enrol. The general principles that guided this website design were that it should be easy to read, use simple language, have a clear format with minimal colour and without distractions, be easy to navigate and quick to load. The CancerHelp UK [36] and the National Cancer Institute [37] websites were identified as consumer-friendly cancer clinical trial websites that were used to guide the development of ACTO.

### ACTO Evaluation

From June 2008 to October 2010 ACTO was the subject of a large cluster randomised trial. Its aim was to evaluate the effect of the website on discussions between patients and medical oncologists about participation in cancer clinical trials and recruitment rates to trials. Thirty medical oncologists from 30 clinics in two Australian states (New South Wales and Victoria) and 493 patients with cancer were recruited (full evaluation data to be reported elsewhere). We conducted a sub-study that recruited patients from one medical oncologist working at a major teaching hospital in Sydney, Australia. This sub-study was completed specifically to collect the views of an unselected sample of cancer patients about the website. These patients were asked to complete an online questionnaire (Q1) which asked demographic questions before their scheduled appointment with their medical oncologist. After completing this questionnaire, all patients were automatically redirected to ACTO. One week after their consultation patients were asked to complete a second online questionnaire (Q2) which asked for reactions to the website. User and hit statistics were obtained by log file analysis using Google Analytics [38].

Statistical analyses were performed using the standard package SAS (version 9, SAS Institute, Cary, NC, USA). The study was approved by of the University of Sydney (Sydney, Australia) and Royal Prince Alfred Hospital (Sydney, Australia).

## Results

### The Australian Cancer Trials website

The components of ACTO that are a result of consumer consultation are described.

**Figure 1:**
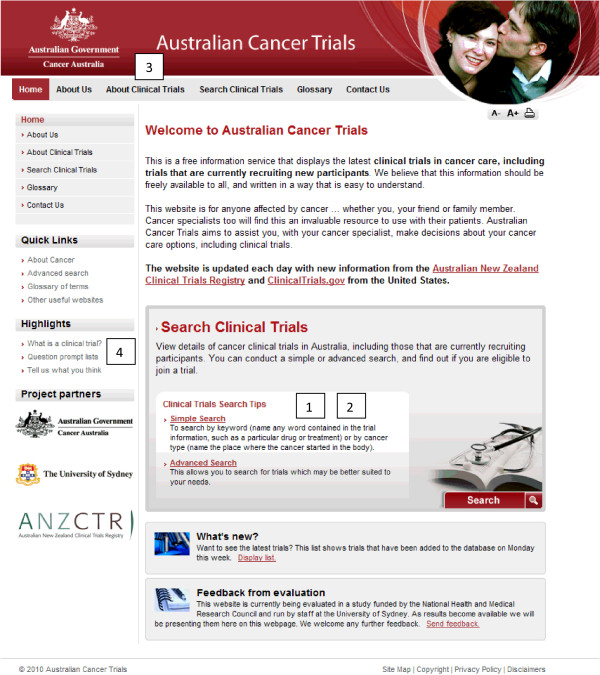
**Home page of the Australian Cancer Trials website**. 1. Search function, 2. Information about each clinical trial (displayed after searching for and then selecting a trial), 3. Supporting information about clinical trial participation "About Clinical Trials", 4. Question prompt lists.

Figure 1 is a screen shot showing the main features of the ACTO home page. These features include 1) A search function; 2) Information about each clinical trial; 3) Supporting information about clinical trial participation; and 4) QPLs.

As guided by consumers, ACTO meets HealthInsite Information Partner requirements [39]. HealthInsite is a federal government initiative which aims to provide access to high quality information about health care to Australians.

#### Source of cancer clinical trial information

Consumers supported the research team's novel idea to use ANZCTR, which registers clinical trials, to automatically download cancer trial information to ACTO. However, it was established that only one-third of cancer trials conducted in Australia are registered with the ANZCTR and approximately two-thirds are registered with the US trial registry, ClinicalTrials.gov (CT.gov). Thus, for ACTO to satisfy consumer demands for a single portal of cancer clinical trials recruiting in Australia, data needed to be sourced from both of these registries.

To achieve this, the content for ACTO is extracted directly from ANZCTR and CT.gov via their data exporting services and downloaded to ACTO on a weekly basis. In contrast, static content (the content which is unchanged week to week) is controlled by Umbraco which is a content management system for publishing content on the Internet. This content is only reviewed and updated occasionally.

#### Cancer trial search function

As of March 2011 1, 040 cancer clinical trials are displayed on ACTO and approximately eight new trials are added every month.

The elements of the search function that consumers identified as important were 1) it should be easy to use even by those without much computer experience; 2) a simple and an advanced search function and; 3) the simple search should have a drop down list of cancer types available.

The simple search page allows users to search by either keyword (eg recurrence, early cancer) or cancer type (using a drop-down list). The advanced search page allows users to refine their search according to cancer stage, phase of trial, recruitment status (open or closed) and location of recruitment.

Consumers requested a facility for users to register for email alerts of newly registered trials or updates about a specific trial. ACTO is working towards providing this service.

#### Information displayed about each clinical trial

The information displayed about each trial on ACTO is shown in Table 1. All of these items were identified as essential by consumers. Most of these details are sourced from routinely collected data provided by trial registrants on ANZCTR and CT.gov.

**Table 1:** Data items about each trial that are displayed on the Australian Cancer Trials website

	Data items
1.	Public title
2.	Recruitment status
3.	Phase of trial
4.	Cancer stage*
5.	Lay summary written by a medical writer*
6.	Description of the study
7.	Target sample size
8.	Primary outcome
9.	Secondary outcomes
10.	Side effects
11.	Inclusion criteria (includes age, gender, health volunteers)
12.	Exclusion criteria
13.	Recruitment dates
14.	Cost and time commitments*
15.	Location of trial*
16.	Ethics approval
17.	Contacts for public and research queries
18.	Trial sponsor(s)
19.	Funding source(s)

*Displayed if a registrant of a cancer trial on the Australian New Zealand Clinical Trials Registry completes the additional cancer items (Table 2).

It is important to note that a lay summary of the trial, cancer stage, anticipated end date for a trial, the cancer treatment type, specific location of recruitment, time, cost and travel commitments and the side effects of the treatments in the trial are not routinely collected by trial registries. To obtain this data, which was additional information requested by consumers, a separate module of "additional cancer items" was developed specifically for registrants of cancer trials on ANZCTR (Table 2). Now, when a cancer trial is registered on ANZCTR, the registrant is encouraged to provide this additional information because it enhances the consumer-friendliness of the trial information when it is displayed on ACTO.

**Table 2:** Additional cancer items collected by Australian New Zealand Clinical Trials Registry from registrants of cancer trials

Data item	Options
Cancer stage	Early
	Locally recurrent or locally advanced
	Metastatic/Widespread
	Not applicable

Treatment type (s)	Treatment: Hormones
	Treatment: Chemotherapy
	Treatment: Targeted therapies and biological therapies
	Treatment: Radiotherapy
	Treatment: Surgery
	Treatment: Other
	Antiemetics
	Complementary
	Palliative care
	Prevention/Screening
	Psychosocial (counselling/training/communication/education)
	Lifestyle
	Rehabilitation

State (s) of recruitment	The Australian state (s) where the participants are being recruited

Anticipated or actual recruitment end date	The anticipated or actual date the trial will stop recruiting patients

Side effects	The known and possible side effect (s) for each trial arm from most to least common

Cost to participants	Similar cost as usual care
	Less cost than usual care
	More cost than usual care

Time commitments	Similar time commitment as usual care
	Less time commitment than usual care
	More time commitment than usual care

Lay summary written by a medical writer	Format of summary:
	• An initial sentence summarizing the trial purpose
	• A section describing the patient eligibility under the heading "Who is it for?"
	• A section entitled "Trial details" which describes the trial design, intervention(s) and comparator(s) and outcome measures of the trial

Consumers felt it was important that the website should include Phase 1, 2 and 3 cancer trials as well as closed trials. Including phase 1 trials was considered particularly important for patients with no further standard treatment options. Closed trials are those closed to recruitment with follow-up continuing, or with completed follow-up. Including closed trials means that patients who enrolled in these trials are still able to see the trial on ACTO, and may enable access to trial results.

In response to the consumer feedback described, all lay summaries have been rewritten by a medical writer who continues to write summaries for new trials as they are registered. Summaries (and the website's supporting information) are written for a reading age of approximately fourteen years.

#### Supporting information about clinical trial participation

Supporting information about clinical trial participation (the "About Clinical Trials section of the website) was informed by consumer recommendations (obtained as described above) and reviewed by the ACTO research team and a medical writer. It includes:

• What clinical trials are and their purpose

• The informed consent process

• Ethical issues

• How a person or a hospital/treatment centre goes about becoming part of a trial

• Assessment issues (eligibility)

• Risks and benefits of participating in a trial

• The process of dissemination of trial results

Permission was obtained from CancerHelp UK to use their glossary for ACTO. Over time, this has been added to and modified.

ACTO contains two QPLs because they were identified as a priority area for consumers at the first NCAG meeting in March 2007 and have been shown to prompt question-asking and increase the likelihood of people having their information needs met [40]. Of the two QPLs developed for the website, one is designed to help consumers ask general questions about clinical trial participation ('General question prompt list: Should I consider joining a clinical trial', Table 3) and the second is a specific QPL called "Should I consider joining this clinical trial?" Both reflect consumer recommended questions and aim to help consumers ask specific questions about a trial that is relevant to their current clinical circumstances. There is a link to the general QPL on the home page of ACTO. A link to the specific QPL is provided next to each clinical trial ("What questions do you need to ask about this clinical trial?"). Hyperlinks are provided from each question to areas on the website that help to answer each question. The QPLs can be printed. Consumers are advised to discuss the QPLs with their cancer specialist.

**Table 3:** General question prompt list: should I consider joining a clinical trial?

Question category	Questions
Understanding my treatment choices	1. What is the usual (standard) treatment for my condition? 2. How do I find a suitable clinical trial? 3. Who can join? 4. How do I take part? 5. How will my confidentiality be protected?

Finding out about a trial	1. How can I learn more about the trial which interests me?

Understanding the possible benefits	1. What benefits could I get by joining a trial? 2. If I join a trial, how might others benefit?

Understanding the possible risks	1. What are the general risks of being in a trial? 2. If I get a side effect or injury because of being in the trial, will I get compensation?

The differences between going on the trial and having standard treatment	1. What are the usual costs of being in a trial and how does this differ from standard treatment?

Types of clinical trials and understanding 'randomisation' and 'blinding'	1. What types of clinical trials are there? 2. What does randomisation mean? 3. What does blinding mean?

Understanding my right to join or not to join the trial	1. Will my doctor still treat me if I decide not to go on a trial? 2. Do I have time to think about whether to go on a trial (a day or two, or a week?) 3. If I join the trial, but later change my mind, how can I stop? 4. Will I be penalised in any way?

Results of the trial	1. How will I be informed of the results of a trial? 2. How are the results measured?

Underlined statements indicate hyperlinks to enable website-users to find the answers to these questions on the Australian Cancer Trials website

#### Website access

ACTO is currently hosted by CA. Consumer organisations are playing an integral role in the promotion and dissemination of ACTO through consumer newsletters and website links, email alerts, consumer organisation forums and multi-disciplinary conferences.

### Australian Cancer Trials website evaluation

#### Access statistics

To comply with the trial protocol the site was not promoted during the trial period from December 2008 until October 2010 so access statistics are reported from 1 November 2010 until 28 February 2011. 2, 549 unique visitors during this period visited the website a total of 3, 342 times ACTO and generated 17, 833 page views. There were an average of 5 pages viewed at each visit and the mean visit duration was 3.4 minutes (range from 1 minute to 13 minutes). A planned promotional program will commence in April 2011 so it expected these numbers will increase.

#### Patient evaluation

The 99 patients in the sub-study had a mean age of 53 years and 70% were female. Twenty-nine percent of patients had breast cancer, 20% had sarcoma, 13% had ovarian cancer and 38% had other cancer types. Of those completing the questionnaires 47 out of 80 (59%) patients responded that they looked at the website. Their feedback about the website is displayed in Table 4. Seventy percent of patients thought the search function was easy to use, 89% thought ACTO was helpful for learning more about cancer clinical trials and all patients thought that it was important for patients to have access to ACTO.

**Table 4:** Sub-study results (n = 47): reactions to the Australian Cancer Trials website

		Number of patients	Percentage
**Rate the following aspects of the website**:			
Searching for cancer clinical trials*			
	Excellent/good	33	70
	Neutral	4	9
	Fair/poor	5	11
			
The information provided about the clinical trials searched, including the lay summary**			
	Excellent/good	22	47
	Neutral	10	21
	Fair/poor	7	15
			
The look, feel and usability of the website***			
	Excellent/good	28	60
	Neutral	12	26
	Fair/poor	3	6
			
The general information about cancer clinical trials***			
	Excellent/good	30	64
	Neutral	8	17
	Fair/poor	5	11
			
**Patients who agreed with the statements**:			
	Helpful for learning more about cancer clinical trials	42/47	89
	Would use Australian Cancer Trials in the future	28/47	60
	Would recommend Australian Cancer Trials to other patients	41/47	87
	Felt it was important for patients with cancer to have access to Australian Cancer Trials	47/47	100

* 5 did not answer ** 8 did not answer *** 4 did not answer

## Discussion

The development of ACTO is an exemplar of a partnership between consumers, clinical researchers and policy makers to create an informative national resource about cancer clinical trials for people affected by cancer, doctors and researchers.

Although they contain valuable information, trial registry websites are not always intuitive to use and are technical in nature. They do not contain related information about clinical trial participation such as a glossary and descriptive information about clinical trial participation. Subsequently, ACTO has been developed using trial registry data, to meet consumers' demands for a comprehensive user-friendly portal of cancer clinical trials recruiting in Australia. It also provides supporting information that can assist them in the decision-making process to join a trial. To be comprehensive, ACTO obtains trial data from both ANZCTR and CT.gov. Sourcing data directly from these clinical trial registries is a unique feature of ACTO and is unlike CancerHelp UK, and the National Cancer Institute both of which maintain their own databases of trials. This important feature increases the efficiency of the website, minimises costs, avoids duplication, and provides good coverage of clinical trials. Furthermore consumers wanted the same source of data to be available to health professionals and the general public. To address all the information consumers considered essential to making a decision about participating in a trial, ANZCTR collects the "additional cancer items" about registered cancer trials especially for ACTO. The Australian Cancer Trials website's features that were informed by consumer-input are summarised in Table 5. Consumer-friendly websites in other health conditions could be developed using a similar model. We note that in 2011 the National Institute for Health Research developed the UK Clinical Trials Gateway which provides easy to understand information about clinical trials in all health conditions using CT.gov and the International Standardised Randomised Controlled Number Register [41,42].

**Table 5:** Summary of the Australian Cancer Trials website's features informed by consumer-input

1.	A single portal of cancer clinical trials recruiting in Australia
2.	Design and features of the simple and advanced search pages
3.	Information displayed about each clinical trial including:
	a. Essential items to be used from clinical trial registries (Table 1)
	b. "Additional cancer items" (Table 2)
4.	Assistance by a medical writer to write a lay summary about each trial
5.	Types of cancer clinical trials included: all trial phases, both open and closed trials
6.	Information in the "About Clinical Trials" section
7.	Glossary
8.	Question prompt lists (see Footnote 1 and Table 3)
9.	Website design: easy to read, simple language (reading age of approximately 14 years), clear format, minimal colour, easy to navigate, quick to load

To answer consumer information needs QPLs have been developed to help patients make informed health care choices [40]. Currently, there are no generic question prompt lists to help consumers considering clinical trial participation. ACTO is unique because it offers two question prompt lists for people considering clinical trial participation, one generic and the other specific to the trial in question. These were modelled on a QPL which had been developed by some of the study team members for a large international breast cancer clinical trial [43]. The importance of asking doctors these questions is highlighted on the website.

Worldwide, other cancer clinical trial websites have had consumer input. The evolution and improvement of the NCI's online cancer information has been "user-driven" from the launch of CancerNet in 1995 to the development of the current NCI website [14]. The NCI has used multiple approaches to gathering input including stakeholder meetings, focus groups, online user surveys, usability testing and search log analysis. Both the NCI website and CancerHelp UK have recently upgraded their websites based on consumer feedback. An advisory board consisting of breast cancer experts and representatives from patient support and advocacy organisations assisted with the development of a German breast cancer trials website [18].

Existing cancer clinical trial websites may have deficiencies. The website content maybe limited, hard to understand and often require users to have high literacy levels [21,22,44]. Websites can be hard to navigate [22]. Online cancer clinical trial search tools may be complex [16] and do not encourage users to consult their doctors. As a result, these websites may not be fulfilling their potential to meet consumers' information needs.

The evidence from our study is that ACTO has overcome these deficiencies. Although the initial evaluation was limited by a small sample, most patients found it easy to search for clinical trials. There appears to be sufficient information on the website. It was encouraging that all respondents thought that it was important for patients to have access to ACTO. People are spending 3.4 minutes on average browsing ACTO, which is similar to the time spent by users of a German breast cancer trials website (mean visit duration 3.2 minutes) [18] and more than the time estimated that most people look at websites [45].

ACTO has some limitations. ANZCTR and CT.gov depend on researchers to provide accurate and up-to-date information about registered trials [46]. Despite being a precondition for publication in an International Committee of Medical Journal Editors journal, not all clinical trials are registered, so it is likely that ACTO does not display every trial that is recruiting in Australia. Furthermore, if the additional cancer items are not provided by registrants of cancer trials on the ANZCTR, the trial will appear on ACTO, but these additional fields will be blank. Similarly not all the additional cancer items can be sourced from CT.gov, highlighting differences in data collection across clinical trial registries. Every effort is being made to encourage registrants to contribute this important information so that it can be made available on ACTO.

Consumer involvement in health care may include participation in research agenda setting [24], evaluating research hypotheses [47], informally or formally evaluating grant proposals [35,48-50], communicating results and supporting the incorporation of research findings into practice [51,52]. The development of ACTO has benefited from consumer involvement in each of these areas: they have helped set the agenda for this initiative, helped obtain research grant funding for the evaluation of ACTO and contributed to the website's development and design. In collaboration with CA, who is continuing to fund the website, consumers led by CVN are playing an essential role in advising about the website's promotion and future enhancements. For example, in collaboration with the research team, they have developed a promotional plan which aims to make the relevant stakeholders aware of ACTO using print media, journal articles, letters, advertisements, posters and bookmarks. Consumers have obtained a more detailed knowledge of trial registries and a closer relationship with the research community as a result of their involvement. The project has enabled opportunities for connections and information exchange between independent consumer organisations (CVN), government organisations (CA and NCAG), ANZCTR and the University of Sydney.

Although the development of a website required technical input, this did not hinder consumer involvement. The research team helped consumers liaise with technical experts so that consumers' desire for easily accessible data about clinical trials could be achieved. In summary, the critical success factors of this project were: (1) that the research team has worked with well-recognised consumer groups with extensive networks; (2) it was determined that a consumer-friendly cancer clinical trials website was a priority for consumer groups and their members and this was supported by the research team; (3) the research team listened to the needs of consumers' and responded to their needs; and (4) the research team assisted consumer groups to provide input when necessary.

## Conclusions

ACTO allows user-friendly access to the information contained in two clinical trials registries, the ANZCTR and CT.gov. Its development is an example of consumers working with doctors, researchers and policy makers to improve the information available to people whose lives are affected by cancer and to help them participate in their treatment decisions, including consideration of clinical trials. Consumer advocacy and input is critical to successful and relevant information resources and has ensured that the website is informative, targets consumer priorities and is user-friendly. ACTO can serve as a model for other health conditions.

## List of abbreviations used

ACTO: Australian Cancer Trials website; ANZCTR: Australian New Zealand Clinical Trials Registry; CA: Cancer Australia; CVN: Cancer Voices New South Wales; CT.gov: ClinicalTrials.gov; NCAG: Cancer Australia's National Consumer Advisory Group; QPL: Question prompt list; SC: Sally Crossing. *Footnote 1: A question prompt list is a structured list of questions for the patient, or their caregiver, to ask their doctor (or other health professional), if they wish.

## Competing interests

The authors declare that they have no competing interests.

## Authors' contributions

RFD made the major contribution to the manuscript's concept and design, coordinated the project to develop the website, conducted the acquisition, analysis and interpretation of data and drafted the manuscript. AB, SC and MT contributed to the manuscript's concept and all authors revised the manuscript critically. All authors have given approval of the final manuscript.

## Authors' information

SC is the Chair of Cancer Voices NSW and Founding Chair of Breast Cancer Action Group NSW. Both groups provide a voice for people affected by cancer wherever decisions impacting them and their treatment are made, for example in government, health service provision, research, support and care services. Since 2000 Cancer Voices NSW has provided the independent voice of people affected by cancer and is active in the areas of diagnosis, information, treatment, research, support and care, working in partnership with providers of these services, ensuring the patient perspective is heard. SH is the national manager for consumer support at Cancer Australia. Cancer Australia was established by the Australian Government to guide improvements in cancer services and research in Australia. Cancer Australia's National Consumer Advisory Group's priorities are to improve consumer information about cancer services, facilitate involvement and choice in treatment options and strengthen quality support for people affected by cancer. RFD, PB, AB and MT are researchers with the Centre for Medical Psychology and Evidence-Based Decision-Making (CeMPED) at the University of Sydney. CeMPED supports research to answer questions about ways to assist patients to be more involved in their own health care.
